# Relationship of Gender With Laser Retinopexy for Retinal Breaks

**DOI:** 10.7759/cureus.39714

**Published:** 2023-05-30

**Authors:** Syed Zohaib Maroof Hussain, Irfan Jeeva, M. A. Rehman Siddiqui

**Affiliations:** 1 Ear, Nose and Throat, Leicester Royal Infirmary, Leicester, GBR; 2 Ophthalmology, Aga Khan University Hospital, Karachi, PAK; 3 Ophthalmology and Visual Sciences, Aga Khan University, Karachi, PAK

**Keywords:** health seeking behavior, gender bias, retinal detachment, retinal breaks, laser retinopexy

## Abstract

Purpose: To explore the relationship of gender with laser retinopexy for retinal breaks in the Pakistani population.

Materials and methods: This was a 10-year retrospective observational study conducted at Aga Khan University Hospital, Karachi, Pakistan. All consecutive patients who underwent laser retinopexy between January 2009 and December 2018 for a retinal tear or high-risk retinal degeneration (such as lattice degeneration) were included in this study. Data were collected from patients’ files. Index eyes with a history of or treatment for retinal detachment were excluded. A structured pro forma was used to collect information. Descriptive statistics were used to explore the relationship between gender and laser retinopexy.

Results: We identified 12,457 patients through the coding system of our hospital who underwent various laser procedures from January 2009 to December 2018. Yttrium aluminium garnet (YAG) laser, laser peripheral iridotomy (PI), and laser trabeculoplasty procedures were all excluded. A total of 3,472 patients’ files were reviewed for this study, out of which 958 patients met the inclusion criteria. Males accounted for a higher number (n=515, 53.87%). The mean age was 43.99±15.37 years. For exploratory analysis, participants were divided into five age groups: <30 years (24.16%); 31-40 years (16.59%); 41-50 years (19.45%); 51-60 years (26.40%); and >60 years (13.49%). Bilateral laser retinopexy was performed in 48.12% of patients; 24.79% and 27.13% of patients underwent unilateral laser retinopexy for the right and left eyes, respectively.

Conclusion: In our cohort study, laser retinopexy was more commonly performed in men than in women. The ratio was not significantly different from the prevalence of retinal tears and retinal detachment in the general population, which has a slightly higher male preponderance. We did not find evidence of significant gender bias among patients who underwent laser retinopexy in our study.

## Introduction

A retinal tear is a full-thickness break in the retina that, if left untreated, may lead to rhegmatogenous retinal detachment (RRD) [1]. The majority of retinal tear formation is the result of posterior vitreous detachment [2]. Prompt treatment of retinal breaks can prevent RRD and significant visual loss. Laser retinopexy is a safe and effective procedure for treatment of retinal breaks [3]. The main aim of laser retinopexy is to prevent RRD [3], which occurs in approximately 1 in 10,000 people/year [2].

Over the last decade, many studies have been conducted to explore the relationship between gender and the treatment of various diseases. Many factors, including biology and social and financial background, contribute to the gender disparity in terms of access to health care. Previously, a major burden was put only on biological differences alone as the factors responsible for disparities. It was assumed that gender and sex were interchangeable terms. Currently, it has been reported that gender differences are not only biological disparities; in fact, they include socioeconomic status, literacy, and male dominance [4].

As reported by a review conducted in developing countries, cataract services are underutilized by women [5]. Although oestrogen has a protective effect against cataracts, two-thirds of all blindness related to cataracts is found in women [4]. Similarly, in Pakistan, blindness due to cataracts is calculated to be twice as common among women than men [6]. One study reported that if females underwent cataract surgery at the same rate as males in developing countries, then the average frequency of cataract-related blindness would be reduced by 12.5% [7].

Another study carried out in the Karachi marine fishing community showed a higher frequency of cataract surgical coverage for females. However, it was reported that their ratio of successful surgery was 4.38 times less than that of males [8].

In contrast, retinal tears have been reported to occur more often in men, usually in the right eye, possibly related to greater average axial length [2,9]. However, at the same time, studies among middle-class Caucasian people have shown men to be reluctant to seek medical help due to traditional masculine behaviour [9].

To the best of our knowledge, there are no data available that explore the relationship between gender and eye health care in Pakistan. The aim of this study was to review disparities in eye health care in the Pakistani population.

This article was previously presented as a meeting abstract at the Association of Surgeons in Training (ASiT) Innovation Summit 2022 on 15-16 November, 2022.

## Materials and methods

This was a retrospective observational study conducted at the Department of Ophthalmology, Aga Khan University Hospital (AKUH), Karachi, Pakistan. All patients who underwent laser retinopexy as the treatment for retinal breaks at our hospital between 1 January 2009 and 31 December 2018 were included. All patients were identified from coding used for laser retinopexy. These codes included codes for laser retinopexy, 360° laser, panretinal photocoagulation (PRP) laser, focal laser and prophylactic laser procedures. The data were collected over a period of four months (March 2019 to June 2019). This study included people of both genders, and there was no age limit. We excluded patients who developed full retinal detachment prior to receiving laser retinopexy and those who underwent other procedures (vitrectomy, pneumaticretinopexy, etc.) for retinal breaks.

Laser procedure was performed after pupil dilation with tropicamide and cyclopentolate 1% eye drops. Drops were repeated three times to achieve maximum dilation as standard. Where pupil dilation was inadequate with standard regimen, the drops were repeated further. Topical anaesthesia with propracaine hydrochloride 0.5% (Alcaine; Alcon, Fort Worth, TX, USA) was performed. Ocular Mainster PRP 165 lens (Ocular Instruments, Bellevue, WA, USA) was used for the laser. Purepoint laser system (Alcon) was used to perform the laser treatment. Laser power was adjusted to achieve a faint white blanching of retina.

The data were collected by using a pro forma in which information was entered from the patients' files. Therefore, informed consent (verbal or written) was not sought. The information that was collected included a general history of the patient (age and gender), ocular history (history of presenting complaints, spherical equivalent, cataracts, uveitis, and yttrium aluminium garnet (YAG) capsulotomy), medical history (diabetes and hypertension), history of trauma to the affected eye and family history related to retinal breaks. During this study, patient privacy and confidentiality were maintained. The Statistical Package for the Social Sciences (SPSS) version 23.0 (IBM Corp., Armonk, NY, USA) was used for data entry and statistical analysis.

Descriptive statistics are reported. Frequencies and percentages (%) are reported for categorical variables (gender, laser retinopathy, initially affected eye, initial symptoms reported, comorbidities, impaired vision, spherical equivalent, family history, trauma to the eye, other eye diseases, medical and surgical history) and assessed by chi-square test where appropriate. The mean ± standard deviation (SD) was reported for quantitative variables such as age. A p value of < 0.05 was considered significant throughout the analysis. This was a retrospective study; therefore, an a priori sample size calculation was not performed.

Ethical approval was obtained for this study from the Ethical Review Committee (ERC) of AKUH (Ref: 2019-0906-2422). This study was conducted in accordance with the tenets of the Declaration of Helsinki.

## Results

**Figure 1:**
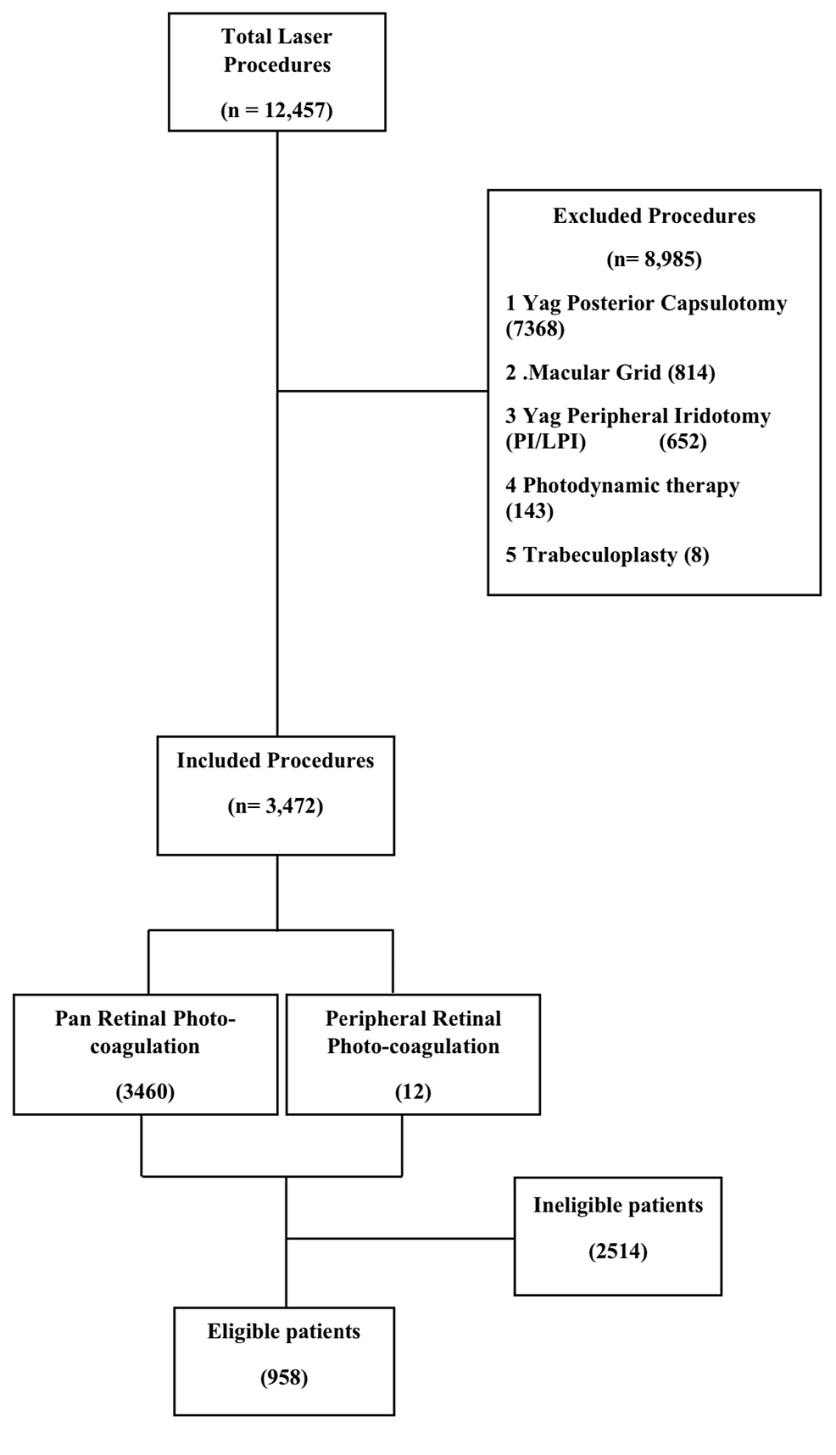
Flow chart illustration of data Yag: yttrium aluminium garnet

We identified 12,457 patients who underwent laser retinopexy procedures between 1 January 2009 and 31 December 2018. Of these, 8,985 patients who underwent other types of laser procedures were excluded. A total of 3,472 patient files were reviewed for this study, out of which 958 patients met the inclusion criteria (Figure 1). Out of a total of 958 eligible patients, the proportion of males was higher, i.e., 53.87% (95% confidence interval (CI), 50.5%-57.0%). For exploratory analysis, participants were divided into five age groups, as shown in Table 1. The mean age was 43.99+15.37 years.

**Table 1:** Sociodemographic characteristics (n=958)

Gender	Frequency	Percentage
Male	515	53.8%
Female	443	46.2%
Age (years) (Mean=43.99+15.37)		
<30	231	24.1%
31-40	159	16.6%
41-50	186	19.4%
51-60	253	26.4%
>60	129	13.5%

Most of the patients presented with blurred vision (45.3%) along with other symptoms. Among individuals who underwent laser retinopexy, bilateral laser retinopexy was performed more commonly (48.1%), as shown in Table 2. Comorbidities and ocular history are reported in Table 3. Comparing the gender distribution with different variables, a significant association was found with the laterality of the affected eye (p<0.001), symptoms (p=0.033), cataract surgery (p=0.041), and trauma (p=0.022), as shown in Table 4.

**Table 2:** Presenting complaints (n=958)

Affected eye	Frequency	Percentage
Both eyes	461	48.1%
Right eye	237	24.7%
Left eye	260	27.1%
Symptoms		
Blurred vision	484	45.3%
Reduced vision	208	19.5%
None	157	14.7%
Floaters	129	12.1%
Flashes	88	8.2%
Others	2	0.2%

**Table 3:** Ocular and general history (n=958)

	Frequency	Percentage
Cataract surgery		
No	515	53.8%
Yes	443	46.2%
Yttrium aluminium garnet (YAG) laser capsulotomy		
No	790	82.5%
Yes	168	17.5%
Diabetes		
No	829	86.5%
Yes	129	13.5%
Uveitis		
No	953	99.5%
Yes	5	0.5%
Hypertension		
No	744	77.7%
Yes	214	22.3%
Trauma		
No	930	97.1%
Yes	28	2.9%
Family History		
No	935	97.6%
Yes	23	2.4%

**Table 4:** Association of sex differences with different variables (n=958)

		Sex		Total	p Value
		Male (n=515)	Female (n=443)		
Affected Eye					0.000
Right eye		153	84	237	
Left eye		136	124	260	
Both		226	235	461	
Age					0.366
<30		119	112	231	
31-40		77	82	159	
41-50		102	84	186	
51-60		147	106	253	
>60		70	59	129	
Symptoms					0.033
Blurred vision		92	65	157	
Reduced vision		58	71	129	
None		248	236	484	
Floaters		41	47	88	
Flashes		123	85	208	
Others		0	2	2	
History					
Cataract surgery	No	263	252	515	0.041
	Yes	252	191	443	
Yttrium aluminium garnet (YAG) laser capsulotomy	No	418	372	790	0.255
	Yes	97	71	168	
Trauma	No	494	436	930	0.022
	Yes	21	7	28	
Diabetes	No	439	390	829	0.207
	Yes	76	53	129	
Hypertension	No	406	338	744	0.347
	Yes	109	105	214	
Uveitis	No	512	441	953	0.779
	Yes	3	2	5	
Family history	No	500	435	935	0.265
	Yes	15	8	23	

## Discussion

This study aimed to review the relationship of health disparities in eye health care and gender in the Pakistani population. Out of 958 eligible patients, a higher number of men (n=515; 53.87%) underwent laser procedures for retinal breaks than women (n=443; 46.24%). Our findings are consistent with those of other epidemiologic studies on retinal tears and retinal detachment [9-11].

Poulsen et al. conducted a study over a period of three years and found that the majority of patients who underwent surgery for RRD were men. They reported male gender as a risk factor for RRD [10]. Similarly, another review reported male preponderance (13.09 vs. 7.41 per 100,000 population) in RRD with longer axial length being the possible cause [11]. In addition to longer axial length, differences in basal vitreoretinal adhesion in males may predispose them to retinal tears, which then lead to RRD [12]. Other risk factors for retinal tears include myopia, aphakia, inflammatory conditions or any trauma. In a review, it was reported that retinal tears were associated with old age [13].

The word gender in relation to health care services was first introduced globally at the Alma Ata Conference in 1978. The theme was “Health for All” [14]. We believe that there are a number of reasons for gender inequality, including structural differences, gender, ethnicity, race, and caste. In a review, it was reported that gender differences will likely persist unless inequalities in availability, affordability, and access to health care are addressed [15].

In Pakistan, particularly in rural areas, access to healthcare services is difficult due to the inconvenient location of healthcare facilities due to the hilly terrain [16], long distances between patients’ homes and healthcare facilities [17], and the lack or inadequate availability of transportation [16]. A lack of human resources at health facilities precludes patients from seeking medical treatment. This includes an insufficient supply of medicines, fewer staff members and/or an absence of female doctors at health facilities [17]. In addition, affordability plays a major role in health care and is measured by occupational status, the husband’s employment, and the household income of women and whether or not they are covered by insurance [16,18].

Our retrospective study was conducted in an urban area of Pakistan with better accessibility and availability of health care. However, in general, accessing health care is influenced predominantly by gender roles in Pakistan [19]. Serving the role of head of household, men also assume the responsibility for making decisions, including regarding women’s health needs and mobility [18]. In addition, various studies have shown that the socioeconomic status of women, permission of the husband and mother-in-law, and lack of health care knowledge influence access to services [18]. Therefore, in a review, it was reported that building medical infrastructure alone will be inadequate to reduce the gender differences in access to health care [20]. In addition to biological differences in health needs, there are socially constructed differences that present an uneven set of opportunities for both men and women [21].

In Pakistan, men seek medical advice more often than females [22]. Additionally, females overlook severity, and they delay medical consultation. In a review conducted in Pakistan, it was reported that more common illnesses among women included diarrhoea/vomiting, joint pain, fever, respiratory and gastrointestinal infections, lethargy, and eye and skin infections. All of the conditions are addressed properly except eye and skin infections [22]. In Pakistan, most of the time, general physicians or optometrists are in charge of eye health. They simply manage eye problems with eyeglasses or eye drops regardless of underlying pathology, such as retinal tears. Likewise, another study conducted in a poor urban community of Karachi found that females were highly neglected in terms of health care. The reasons behind this inequity were multifactorial, including financial constraints, illiteracy, the domestic responsibilities of women and the dominant role of men [23].

Health-seeking behaviour (HSB) is also reflected by the geographical location of women. In comparison to urban areas, more than two-thirds of the population resides in rural areas of Pakistan. A total of 50% of the population is female [22]. Discrimination against women in terms of health, education, nutrition, and rights is a painful reality. This can be linked to the low socioeconomic status of women in rural and remote areas. However, cultural and religious misinterpretations have also placed women’s health in danger [24]. Unfortunately, females are given lower priority than male children starting in childhood [25]. Hasan and Khanum found that boys are taken as twice as often as girls to healthcare providers [26]. This clearly shows that discrimination starts in childhood and prevails in our society [27]. These inequalities are reflected in antenatal and postnatal care as well. As evidenced by a review, only 39% of all deliveries are supervised by skilled birth attendants (doctor, nurse, midwife, or female health worker) in comparison to 65% of deliveries performed at home [26]. These numbers reflect suboptimal care-seeking behaviour during pregnancy among Pakistani women [20]. Furthermore, postpartum care was more neglected than antenatal care (43 vs. 61%, respectively) [27].

Moreover, gender disparities are not uncommon in trauma care; female patients are the victims of negligence in many health settings. However, fewer women are transferred to trauma care for initial triage compared to men, and the majority of women are treated only in nontrauma centres regardless of the severity of the injury. The reasons for this disparity are multifactorial and include health personnel bias, differences in the severity of injury and causes of trauma [28].

Another important reason for the disparity in HSB is economic barriers. Economic instability is a common problem in developing countries. In most developing countries in South Asia, it has been reported that health expenditures are at times as high as out-of-pocket costs can be [29]. This is the same in Pakistan. Total health care costs preclude people from seeking medical care in Pakistan, particularly women, due to their dependency on men [17]. These costs include consultation fees, money spent on medicines, and commuting fares [29]. This leads to people having to pay out of pocket and hence, results in a delay in immediate health care in Pakistan.

The healthcare system is very complicated in Pakistan; therefore, it is crucial for various sectors to plan and work together for better and safe healthcare for the Pakistani population. Thus, it is important to understand the HSBs of the population and the factors driving these behaviours. Formulating strategies to develop overall better health services requires an understanding of the factors behind HSBs in a diverse healthcare system. This applies to both the public and private sectors [30].

Regardless of the sociodemographic status of females, it is important to recruit more female health workers and provide them with a safe environment, good support, and quality care. The present study highlighted the gender disparities among laser retinopexy patients; however, it is important to understand and address the reasons for these disparities. Therefore, policy makers must understand health behaviours and healthcare use at the district level and give sufficient credence to these facts. This will help in designing better health policies. In-depth research is crucial to understand the real picture of the habits and practices of the people in our region [30]. Primarily, the focus should be placed on rural areas and remote areas, and resources should not be given only to large cities such as Karachi and Islamabad [24].

Finally, a difficult and more challenging task is translating research into solid policy plans and actions. Research in this area will definitely impact the direction or implementation of currently launched health programs in Pakistan. It will also help authorities in both the government and private sectors make necessary changes in the administration and redesign of interventions [30].

The strengths of our study include large-scale data from Pakistan, the involvement of multiple surgeons, and one of the largest health sectors in a developing country. The major limitation of our study is its retrospective nature.

## Conclusions

In our study, laser retinopexy was more commonly performed in men than in women. The ratio was not significantly different from the prevalence of retinal tears and retinal detachment in the general population, which has a slightly higher male preponderance. We did not find evidence of significant sex bias among patients who underwent laser retinopexy in our study.
